# Hydrogen Gas Therapy in Schizophrenia: Potential Neuroprotective Effects From an Animal Study

**DOI:** 10.1002/npr2.70117

**Published:** 2026-04-07

**Authors:** Nobumi Miyake, Toshiaki Haga, Shin‐Ichi Hirano, Yusuke Ichikawa, Yoshiyo Oguchi, Kumiko Ando

**Affiliations:** ^1^ Department of Neuropsychiatry St. Marianna University School of Medicine Kawasaki Kanagawa Japan; ^2^ Department of Neuropsychiatry Kawasaki Tama Municipal Hospital Kawasaki Kanagawa Japan; ^3^ Independent Researcher Chigasaki Japan; ^4^ Department of Research and Development MIZ Company Limited Kamakura Kanagawa Japan; ^5^ Department of Student Health Center Institute of Science Tokyo Tokyo Japan

**Keywords:** antioxidant therapy, hydrogen gas, MK‐801, schizophrenia‐like mouse model

## Abstract

**Aims:**

Oxidative stress has recently emerged as a key factor in the pathophysiology of schizophrenia. This study investigated whether inhalation therapy with hydrogen gas, a selective antioxidant, could reduce oxidative stress and improve behavioral outcomes in an MK‐801–induced schizophrenia‐like mouse model.

**Methods:**

Six‐week‐old male C57BL/6 mice received chronic intraperitoneal injections of MK‐801 (0.5 mg/kg) or saline for 4 weeks. A third group received MK‐801 followed by hydrogen gas inhalation therapy. Behavioral assessments included the open‐field test (OFT) to evaluate hyperactivity as an indicator of positive symptoms and the prepulse inhibition (PPI) test to assess cognitive dysfunction. Oxidative stress was evaluated by measuring whole‐brain hydroxyl radical antioxidant capacity (HORAC) and total antioxidant capacity (TAC).

**Results:**

MK‐801–treated mice exhibited significant hyperactivity in the OFT and a trend toward impaired PPI, confirming the schizophrenia‐like phenotype. Hydrogen gas treatment did not produce significant improvements. MK‐801 administration significantly reduced HORAC levels, whereas hydrogen gas therapy markedly restored them. No significant differences in TAC were observed among the groups.

**Conclusion:**

These findings suggest that hydrogen gas therapy does not significantly ameliorate behavioral abnormalities in the MK‐801–induced schizophrenia‐like mouse model but exerts beneficial antioxidant effects. Future studies should evaluate hydrogen gas as an adjunctive therapy and further assess its efficacy and safety in clinical settings.

## Abstract 2

Hydrogen gas inhalation restored antioxidant capacity in a schizophrenia‐like mouse model but did not significantly improve behavioral deficits. These findings suggest that hydrogen may have potential as an adjunct therapy targeting oxidative stress in psychiatric disorders.

## Graphical abstract

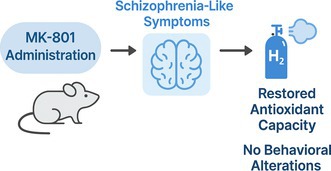

## 1 Introduction

Schizophrenia is a chronic disorder characterized by positive, negative, and cognitive symptoms [1]. Multiple hypotheses have been proposed to explain its pathophysiology, including dopamine [2, 3, 4] and glutamate [5, 6] hypotheses; however, no definitive mechanism or curative treatment has been identified. New‐generation antipsychotics offer only modest advantages over first‐generation drugs and are often associated with safety and tolerability concerns. Consequently, there remains an unmet need for more effective and better‐tolerated therapeutic strategies [7].

Recent evidence highlights oxidative stress as a major contributor to the pathological progression of schizophrenia by exacerbating neuroinflammation and disrupting synaptic function [8, 9, 10]. A meta‐analysis has demonstrated impaired antioxidant defenses in drug‐naïve patients and suggested that antipsychotics alter both enzymatic and nonenzymatic antioxidant systems [11]. However, these effects appear insufficient to address ongoing therapeutic challenges.

MK‐801, an *N*‐methyl‐d‐aspartate (NMDA) receptor antagonist, is widely used to induce schizophrenia‐like behaviors in mice and has been shown to increase oxidative stress. Several antioxidants have demonstrated protective effects against MK‐801–induced changes [12, 13, 14]. Molecular hydrogen has emerged as a potential therapeutic antioxidant in various diseases, including Parkinson's disease and cancer, due to its ability to selectively scavenge hydroxyl radicals and protect cells [15]. This study examined whether hydrogen gas inhalation could reduce oxidative stress and improve behavioral outcomes in an MK‐801–induced schizophrenia‐like mouse model.

## 2 Materials and Methods

### 2.1 Animals

**FIGURE 1:**
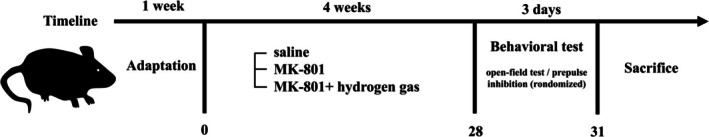
Schematic representation of the experimental design for the MK‐801–induced schizophrenia‐like mouse model.

This study was conducted in accordance with the National Institutes of Health Guide for the Care and Use of Laboratory Animals and was approved by the Institute for Animal Experimentation, St. Marianna University Graduate School of Medicine. Six‐week‐old male C57BL/6 mice were purchased from Japan SLC Inc. (Shizuoka, Japan). Animals were housed in a temperature‐ and humidity‐controlled environment under a 12‐h light/dark cycle with ad libitum access to standard chow and water. After a 1‐week acclimation period, mice were randomly assigned to three groups (7–10 mice per group): saline‐treated controls, MK‐801–treated mice, and MK‐801 plus hydrogen gas‐treated mice. The experimental design is shown in Figure 1.

### 2.2 Drug Administration

MK‐801 (FUJIFILM Wako Pure Chemical Corporation, Osaka, Japan) was dissolved in saline and administered intraperitoneally at a dose of 0.5 mg/kg once daily for 4 weeks. Control mice received an equal volume of saline. The injection protocol was adapted from previous studies with minor modifications [12, 16].

### 2.3 Hydrogen Gas Therapy

Hydrogen gas therapy was administered for 4 weeks following MK‐801 treatment. Mice were placed in a humidified chamber and exposed to 7% hydrogen mixed with air for 90 min per day. Control mice were placed in an identical isomorphic chamber without hydrogen exposure. Hydrogen gas was supplied using a hydrogen gas supply apparatus (MiZ Co., Kanagawa, Japan) at a total flow rate of 2 L/min, with concentrations maintained below the explosive threshold [17]. Internal hydrogen levels were not directly measured, as previous studies using comparable conditions have demonstrated higher hydrogen concentrations in blood and tissues [18, 19].

### 2.4 Behavioral Studies

After 4 weeks of treatment, mice underwent behavioral testing, including the open‐field test (OFT) and prepulse inhibition (PPI) test.

### 2.5 OFT

Locomotor activity was measured using an OFT apparatus (40 × 40 × 45 cm). Each mouse was placed in a corner and allowed to explore freely for 30 min. Ambulatory activity and movement times were analyzed using the SMART Video Tracking System (Panlab, Spain). The software's default immobility settings were used, in which immobility is detected when movement speed remains below 2.5 cm/s for at least 0.5 s. Small movements such as grooming do not exceed this threshold and are therefore classified as resting. Locomotor activity was quantified as total distance traveled and used as an index of hyperlocomotion.

### 2.6 PPI

PPI of the acoustic startle response was measured using a startle chamber (SR‐LAB system; San Diego Instruments, San Diego, CA, USA). Chambers were equipped with an acrylic cylindrical enclosure and a small electric fan generating 60‐dB background noise and ventilation. Broadband noise pulses were delivered through a speaker positioned above the animal, and startle responses (whole body flinch) were detected by an accelerometer attached to the enclosure. The sound pulse parameters were controlled using SR‐LAB software, which also recorded the startle response.

Mice were acclimated to the chamber for 30 min before testing. A continuous 60‐dB white background noise was presented throughout the session. The test consisted of 30 trials with three stimulus conditions: prepulse stimuli (75 dB, 30 ms), white noise (60 dB, 100 ms), and pulse‐alone (120 dB, 30 ms). Each condition was presented 15 times with a 10‐s intertrial interval. PPI (%) was calculated as:

$$\begin{array}{c} \mathrm{PPI}=100–([\text{startle amplitude during prepulse}-\text{pulse trials}/\\ \;\text{startle amplitude during pulse}-\text{alone trials}]\times 100). \end{array}$$

### 2.7 Tissue Preparation and Antioxidant Capacity Measurement

Oxidative stress was evaluated by measuring brain hydroxyl radical antioxidant capacity (HORAC) and total antioxidant capacity (TAC) using commercial assay kits from Cell Biolabs Inc. (San Diego, CA, USA). All reagents were of analytical grade.

Mice were sacrificed by decapitation 1 day after behavioral testing. Brains were immediately removed, rinsed with 0.9% NaCl, weighed, and transferred to thick‐walled glass tubes. Tissues were homogenized (1:10, w/v) in cold phosphate‐buffered saline (pH 7.4) on ice for 10 s at 10‐s intervals. The process was completed after the tissues were fully homogenized. The tissue lysates were centrifuged at 10 000 × g for 10 min. All the procedures were performed at 4°C.

Supernatants were stored at −80°C until analysis. Antioxidant capacities were determined by comparison with standards, such as Gallic Acid Equivalents for HORAC and Copper Reducing Equivalents for TAC, according to the manufacturer's instructions.

### 2.8 Statistical Analysis

Statistical analyses were performed using Statistical Package for the Social Sciences (SPSS), version 27.0. Normality tests were used to assess data distribution. Parametric analysis of variance (ANOVA) was used to compare normally distributed data, followed by Bonferroni correction for multiple comparisons. All tests were two‐tailed, and a *p*‐value < 0.05 was considered statistically significant.

## 3 Result and Discussion

**FIGURE 2:**
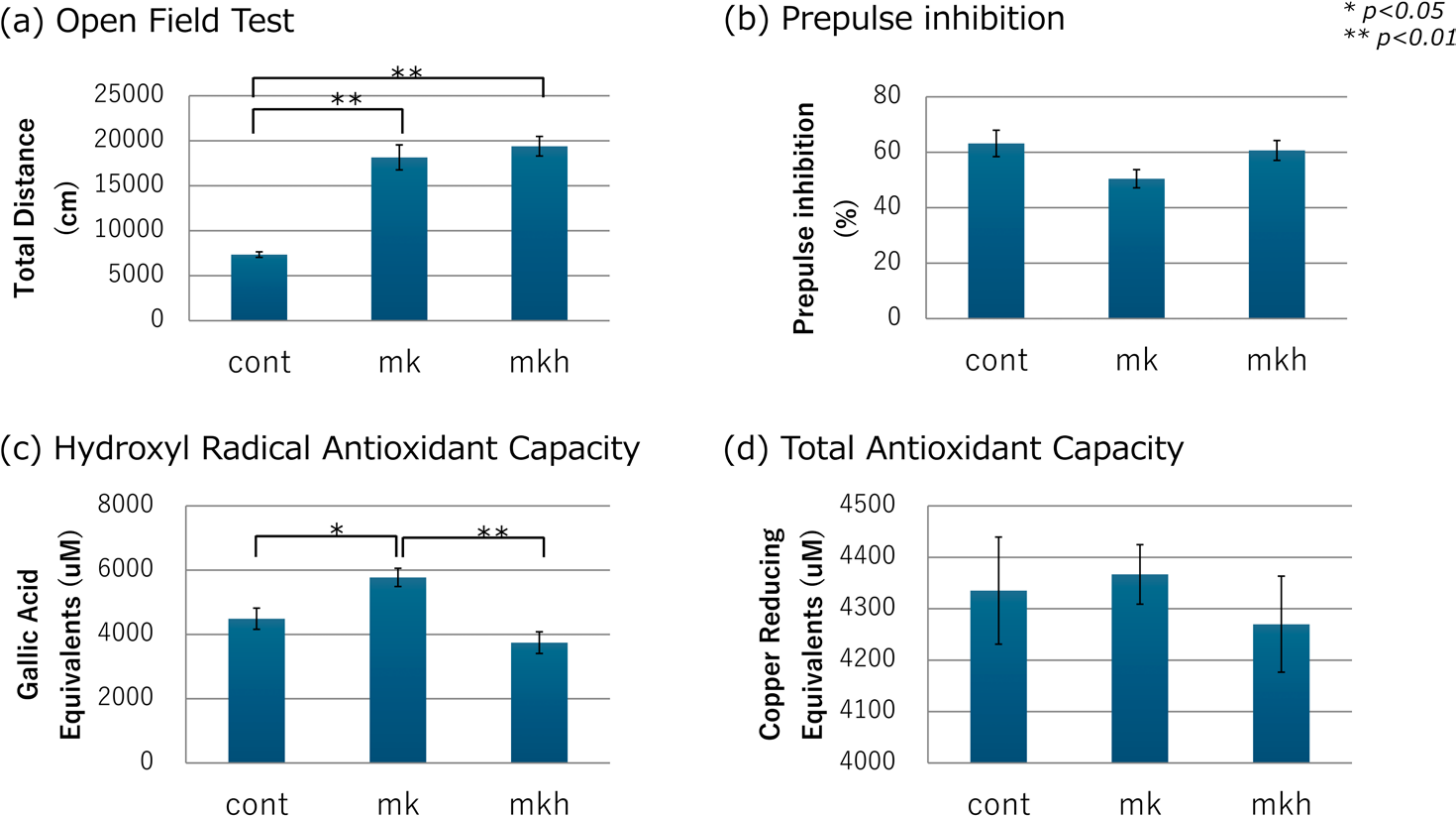
Effects of chronic treatment with saline (cont), MK‐801 (0.5 mg/kg; mk), and MK‐801 combined with hydrogen gas (mkh) on: (a) open‐field test (OFT), (b) prepulse inhibition (PPI), (c) hydroxyl radical antioxidant capacity (HORAC), and (d) total antioxidant capacity (TAC). Data are presented as the mean ± standard error (*n* = 10–11 per group). * *p* < 0.05, ** *p* < 0.01. Statistical significance was determined using one‐way analysis of variance (ANOVA), followed by Bonferroni correction. (a) *F* = 37.316, *p* < 0.001; (b) *F* = 3.050, *p* = 0.063; (c) *F* = 10.767, *p* < 0.001. Oxidative stress caused by MK801, as shown by HORAC, was significantly reduced (*p* < 0.01) by hydrogen gas treatment. (d) *F* = 0.319, *p* = 0.73.

The results are summarized in Figure 2. Compared with saline‐treated controls, MK‐801–treated mice showed significant hyperactivity in the OFT (*p* < 0.01) and a trend toward impaired PPI (*p* = 0.085), supporting the validity of the schizophrenia‐like phenotype. Hydrogen gas therapy did not significantly alter performance in the OFT or PPI. MK‐801 significantly reduced whole‐brain HORAC (*p* < 0.05), whereas hydrogen gas therapy markedly improved these scores (*p* < 0.01). No significant changes were observed in TAC among the groups.

To our knowledge, this is the first study to examine the effects of hydrogen gas therapy in a schizophrenia‐like mouse model. Although hydrogen gas has been reported to ameliorate cognitive impairment and oxidative stress in various disease models and clinical studies [20, 21, 22], our findings indicate that it does not significantly improve behavioral abnormalities in the MK‐801 model. The lack of behavioral effects and cognitive improvements may be attributable to the limited duration of hydrogen gas exposure. Although the OFT results showed significant differences and the PPI data exhibited a positive trend, the relatively modest PPI deficit in the MK‐801 group may have reduced the sensitivity of the treatment to detect effects on schizophrenia‐like phenotypes. In addition, the small sample size limited statistical power, precluding meaningful correlation analyses.

Hydrogen selectively scavenges hydroxyl radicals rather than acting as a broad‐spectrum antioxidant. Because HORAC specifically reflects hydroxyl radical scavenging capacity, whereas TAC represents global antioxidant capacity, the selective improvement in HORAC without changes in TAC is mechanistically plausible. Future studies should examine different doses and durations of hydrogen gas therapy to further clarify its antioxidant potential.

## 4 Future Perspectives

To address the limitations of this study, future work could explore the potential of “Mega‐hydrogen therapy,” involving higher concentrations or longer durations of hydrogen gas exposure. Increasing evidence suggests that such approaches enhance cellular antioxidant capacity and reduce oxidative stress in various disease models [15]. Additionally, studies using alternative schizophrenia models, including genetically modified animals, are warranted.

Although hydrogen therapy inhibits oxidative stress, it does not directly modulate dopaminergic hyperactivity, which may explain its ineffectiveness against positive symptoms. Therefore, hydrogen gas may be more suitable as an adjunctive therapy combined with established antipsychotic medications. This combined approach may enhance treatment efficacy and improve clinical outcomes in patients with psychiatric disorders.

Larger, more systematic preclinical studies are required to establish its efficacy and safety and to inform potential clinical applications.

## Author Contributions

N.M. and T.H. had full access to all study data and took responsibility for data integrity and accuracy of the analyses. N.M. and T.H. contributed to study conception and design, performed the experiments, and conducted statistical analyses. All authors contributed to data acquisition, interpretation, and manuscript writing. S.‐I.H., Y.I., Y.O., and K.A. supervised the study and reviewed the manuscript.

## Funding

The authors have nothing to report.

## Conflicts of Interest

Dr. Miyake received speaker honoraria from Boehringer Ingelheim, Eisai, Meiji, Otsuka, Sumitomo, Takeda, and Viatris. Dr. Oguchi received speaker honoraria from Daiichi Sankyo, Eisai, Janssen, Kyowa, Otsuka, Sumitomo, Takeda, Lundbeck, Meiji, Mochida, MSD, and Viatris, as well as a scholarship from Eisai. Dr. Ando received speaker honoraria from Sumitomo and Takeda. Dr. Hirano is an employee of MiZ Co. and the developer and manufacturer of the hydrogen gas supply apparatus used in this study. Dr. Ichikawa is an employee of MiZ Co. The other authors declare no conflicts of interest.

## Supporting information


**Data S1:** npr270117‐sup‐0001‐DataS1.xlsx.

**Data S2:** npr270117‐sup‐0002‐DataS2.xls.

**Data S3:** npr270117‐sup‐0003‐DataS3.xls.

**Data S4:** npr270117‐sup‐0004‐DataS4.xls.

**Data S5:** npr270117‐sup‐0005‐DataS5.xls.

**Data S6:** npr270117‐sup‐0006‐DataS6.xls.

**Data S7:** npr270117‐sup‐0007‐DataS7.xls.

**Figure S1:** Total resting time (%) in the Open Field Test.
**Table S1:** All data used for analysis.
**Table S2:** Raw data for the control group (cont1–5).
**Table S3:** Raw data for the mk group (mk1–6).
**Table S4:** Raw data for the mkh group (mkh1–5).
**Table S5:** Raw data for the control group (cont6–10).
**Table S6:** Raw data for the mkh group (mkh6–11).
**Table S7:** Raw data for the mk group (mk7–11).

## Data Availability

The data that support the findings of this study are available on request from the corresponding author. The data are not publicly available due to privacy or ethical restrictions.
