# Supplementary material for: Hydrogen Gas Therapy in Schizophrenia: Potential Neuroprotective Effects From an Animal Study
Source: Neuropsychopharmacol Rep. 2026 Apr 7;46(2):e70117. doi: 10.1002/npr2.70117 (PMC13054952; doi:10.1002/npr2.70117)
Supplement: Supplementary file 8 — Figure S1: Total resting time (%) in the Open Field Test. Table S1: All data used for analysis. Table S2: Raw data for the control group (cont1–5). Table S3: Raw data for the mk group (mk1–6). Table S4: Raw data for the mkh group (mkh1–5). Table S5: Raw data for the control group (cont6–10). Table S6: Raw data for the mkh group (mkh6–11). Table S7: Raw data for the mk group (mk7–11). [file NPR2-46-e70117-s001.zip › npr270117-sup-0009-TableS1-S7-FigureS1@Supplementary_Figure_and_Table_Captions2.docx]

Supplementary Table 1. All data used for analysis.

File name: All data for analysis.xlsx

This Excel file contains the complete dataset used for statistical analyses.

Supplementary Table 2. Raw data for the control group (cont1–5).

File names: OF 20231208 cont1-5.xlsx

This Excel file contains the unprocessed raw tracking data for the control group (cont1–5), exported directly from the SMART Video Tracking System. The dataset includes time‑stamped positional coordinates and movement trajectories for each mouse during the Open Field Test.

Supplementary Table 3. Raw data for the mk group (mk1–6).

File names: OF 20231210 MK1-6.xlsx

This Excel file contains the unprocessed raw tracking data for the mk group (mk1–6), exported directly from the SMART Video Tracking System. The dataset includes time‑stamped positional coordinates and movement trajectories for each mouse during the Open Field Test.

Supplementary Table 4. Raw data for the mkh group (mkh1–5).

File names: OF 20231211 MKH1-5.xlsx

This Excel file contains the unprocessed raw tracking data for the mkh group (mkh1–5), exported directly from the SMART Video Tracking System. The dataset includes time‑stamped positional coordinates and movement trajectories for each mouse during the Open Field Test.

Supplementary Table 5. Raw data for the control group (cont6–10).

File names: OF 20231208 cont6-10.xlsx

This Excel file contains the unprocessed raw tracking data for the control group (cont6–10), exported directly from the SMART Video Tracking System. The dataset includes time‑stamped positional coordinates and movement trajectories for each mouse during the Open Field Test.

Supplementary Table 6. Raw data for the mkh group (mkh6–11).

File names: OF 20231210 MKH6-11.xlsx

This Excel file contains the unprocessed raw tracking data for the mkh group (mkh6–11), exported directly from the SMART Video Tracking System. The dataset includes time‑stamped positional coordinates and movement trajectories for each mouse during the Open Field Test.

Supplementary Table 7. Raw data for the mk group (mk7–11).

File names: OF 20231211 MK7-11.xlsx

This Excel file contains the unprocessed raw tracking data for the mk group (mk7–11), exported directly from the SMART Video Tracking System. The dataset includes time‑stamped positional coordinates and movement trajectories for each mouse during the Open Field Test.

Supplementary Figure 1. Total resting time (%) in the Open Field Test.

This figure shows the mean ± SEM resting time for the control (cont), mk, and mkh groups. Significant differences were determined using appropriate statistical tests (**p < 0.01).
