# Supplementary figures and images for: Hydrogen Gas Therapy in Schizophrenia: Potential Neuroprotective Effects From an Animal Study
Source: Neuropsychopharmacol Rep. 2026 Apr 7;46(2):e70117. doi: 10.1002/npr2.70117 (PMC13054952; doi:10.1002/npr2.70117)

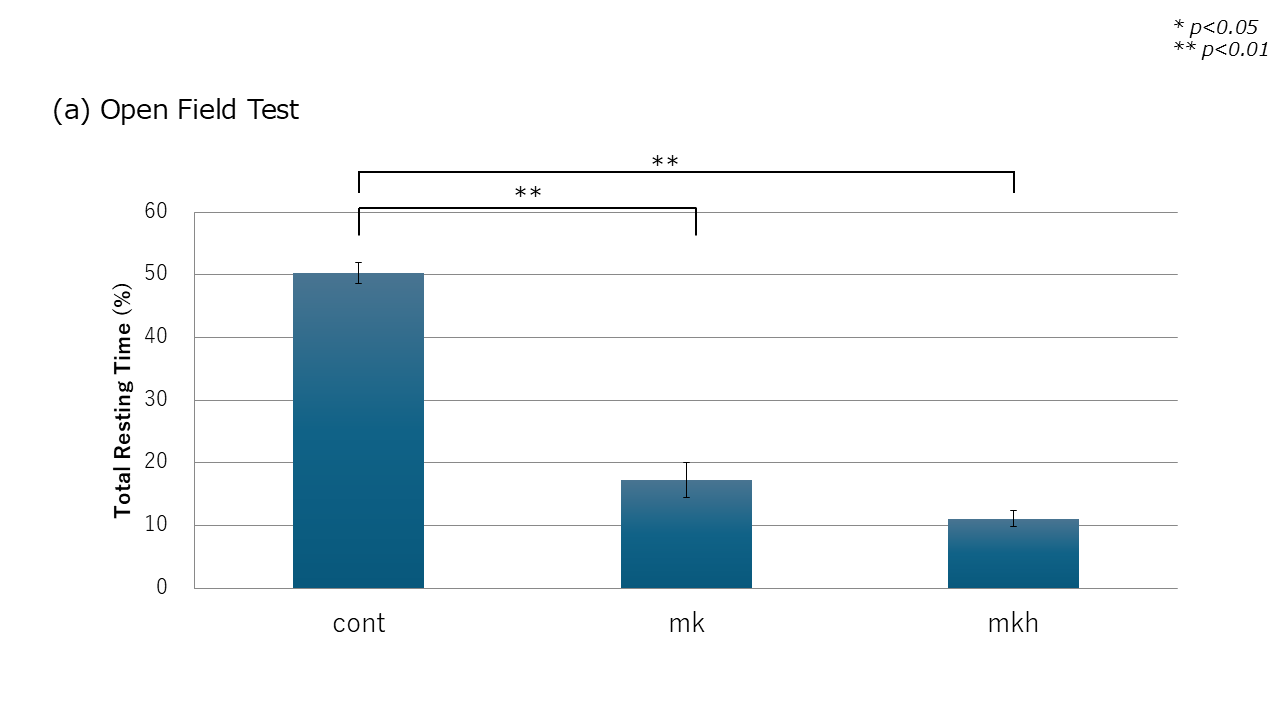

Supplement: Supplementary file 8 — Figure S1: Total resting time (%) in the Open Field Test. Table S1: All data used for analysis. Table S2: Raw data for the control group (cont1–5). Table S3: Raw data for the mk group (mk1–6). Table S4: Raw data for the mkh group (mkh1–5). Table S5: Raw data for the control group (cont6–10). Table S6: Raw data for the mkh group (mkh6–11). Table S7: Raw data for the mk group (mk7–11). [file NPR2-46-e70117-s001.zip › npr270117-sup-0008-FigureS1@Supplementary figure1.tif]
